# *In Vitro* Response of Retinal Pigment Epithelial Cells Exposed to Chitosan Materials Prepared with Different Cross-Linkers

**DOI:** 10.3390/ijms11125256

**Published:** 2010-12-20

**Authors:** Jui-Yang Lai, Ya-Ting Li, Tsu-Pin Wang

**Affiliations:** 1 Institute of Biochemical and Biomedical Engineering, Chang Gung University, Taoyuan, 33302, Taiwan; 2 Biomedical Engineering Research Center, Chang Gung University, Taoyuan, 33302, Taiwan; 3 Molecular Medicine Research Center, Chang Gung University, Taoyuan, 33302, Taiwan

**Keywords:** chitosan, cross-linking, genipin, glutaraldehyde, cytocompatibility, retinal pigment epithelial cells

## Abstract

The interaction between cells and biopolymers is the evaluation indicator of the biocompatibility of materials. The purpose of this work was to examine the responses of retinal pigment epithelial (RPE) cells to genipin (GP) or glutaraldehyde (GTA) cross-linked chitosan by means of cell viability assays, cytokine expression analyses, and apoptosis assays. Evaluations of non-cross-linked chitosan were conducted simultaneously for comparison. Both GP and GTA treated samples with the same extent of cross-linking (around 80%) were prepared by varying cross-linking time. Our results showed that GP cross-linking was carried out by either radical polymerization of the monomers or S_N_2 nucleophilic substitution reaction involving the replacement of the ester group on the monomer with a secondary amide linkage. On the other hand, GTA could react with free amino groups of chitosan, leading to the formation of either the Schiff bases or the Michael-type adducts with terminal aldehydes. The biocompatibility of non-cross-linked chitosan membranes was demonstrated by the absence of any signs of toxicity or inflammation reaction. The present study showed that the ARPE-19 cells exposed to GTA cross-linked chitosan membranes had significantly higher cytotoxicity, interleukin-6 levels, and number of TUNEL-positive nuclei than did those exposed to GP treated samples. In addition, the materials modified with GTA trigger apoptosis at an early stage and may induce toxicity in the RPE cells later. The findings suggest that while the chitosan molecules bridged by GP are satisfactorily cytocompatible, the counterparts treated by GTA do not seem to be tolerated. In terms of material safety, the GP cross-linked chitosan may be compatible with human RPE cells and may have a potential application as delivery carriers in the treatment of posterior segment diseases.

## 1. Introduction

Age-related macular degeneration (AMD) is a clinical entity well known in people 50 years of age or older in the developed world [1]. Retinal transplantation has been investigated as a possible therapeutic strategy for repair of damaged tissues in such degenerative conditions [2–4]. However, the limited availability of donor retinas is a major barrier in transplantation medicine. Currently, other techniques such as pharmacologic therapy and gene therapy are being studied to treat AMD. In 2005, Kataoka *et al.* [5] developed a polyion complex micelle that can be used as a novel delivery carrier for dendrimer porphyrin. The highly selective accumulation of the micelle-encapsulated dendrimer porphyrin into choroidal neovascularization lesions may result in a significantly pronounced photodynamic effect with minimal unfavorable phototoxicity. Their group also presents a ternary complex system composed of a core containing DNA packaged with cationic peptides and enveloped in the anionic dendrimer phthalocyanine, which exhibits more than 100-fold photochemical enhancement of transgene expression *in vitro* with reduced photocytotoxicity [6]. These studies suggest that by means of electrostatic interaction with oppositely charged carrier materials, the negatively charged drug or DNA can stably be incorporated into the nanocarrier for more effective delivery.

Chitosan is a naturally occurring cationic polysaccharide obtained by deacetylation of chitin, a biopolymer second in abundance to cellulose. It is primarily comprised of repeating d-glucosamine units and shows antibacterial and wound healing activities [7,8]. As a biomaterial, chitosan has good biocompatibility [9,10]. In the field of ophthalmology, chitosan has been of great interest in drug delivery applications. The mucoadhesiveness of chitosan makes it an attractive carrier for increasing corneal residence time and enhancing bioavailability by interacting with the negative charges of the mucus [11]. A recent review by Alonso *et al.* [12] has provided a detailed description of the potential of chitosan-based nanosystems for topical ocular administration. Despite numerous studies supporting the benefits of chitosan in delivering drugs onto the eye surface, very limited experience on the intraocular use of chitosan carriers has been reported [12].

Cross-linking is recommended to overcome the hydrophilic nature of chitosan for its application as a drug carrier [9]. Water-soluble carbodiimides are considered as non-toxic cross-linkers because they can be found as by-products of the reaction, in the form of urea derivatives [13]. In our laboratory, carbodiimides have been successfully applied for cross-linking of biopolymers such as gelatin [4,14] and hyaluronic acid [15,16] to fabricate cell/tissue delivery carriers. Since, in essence, chitosan materials carry free amine functionalities and lack free carboxylic acid groups available for carbodiimide cross-linking, other agents, *i.e.*, genipin (GP) and glutaraldehyde (GTA) are more feasible for chemical modification. It is known that although cross-linking is a common method to increase the stability of biomaterials, the treatment involving the use of chemical cross-linkers probably causes toxic side-effects [17]. Several studies have evaluated the compatibility of GP or GTA cross-linked chitosan-based materials towards HeLa cells [18], human foreskin fibroblasts [19], L929 murine fibroblasts [20], Caco-2 cells [21], and pig iliac endothelial cells [22]. However, the previous documents do not have the focus on the ocular biocompatibility of chemically modified chitosan.

In view of the potential of the development of cross-linked chitosan-based drug delivery systems for the treatment of posterior segment diseases such as AMD, diabetic retinopathy, and cytomegalovirus retinitis, there must be concern about the possible safety issues related to chemical cross-linking of biomaterials. Here, we performed an *in vitro* study to examine the responses of retinal pigment epithelial (RPE) cells to GP or GTA treated chitosan. Evaluations of non-cross-linked chitosan samples were conducted simultaneously for comparison. The information from cell-material interactions that is relevant to ocular biocompatibility will facilitate the engineering of the appropriate cross-linked chitosan to enhance the delivery of drug therapeutics.

## 2. Results and Discussion

### 2.1. Preparation of GP or GTA Cross-Linked Chitosan Membranes

**Figure 1.**
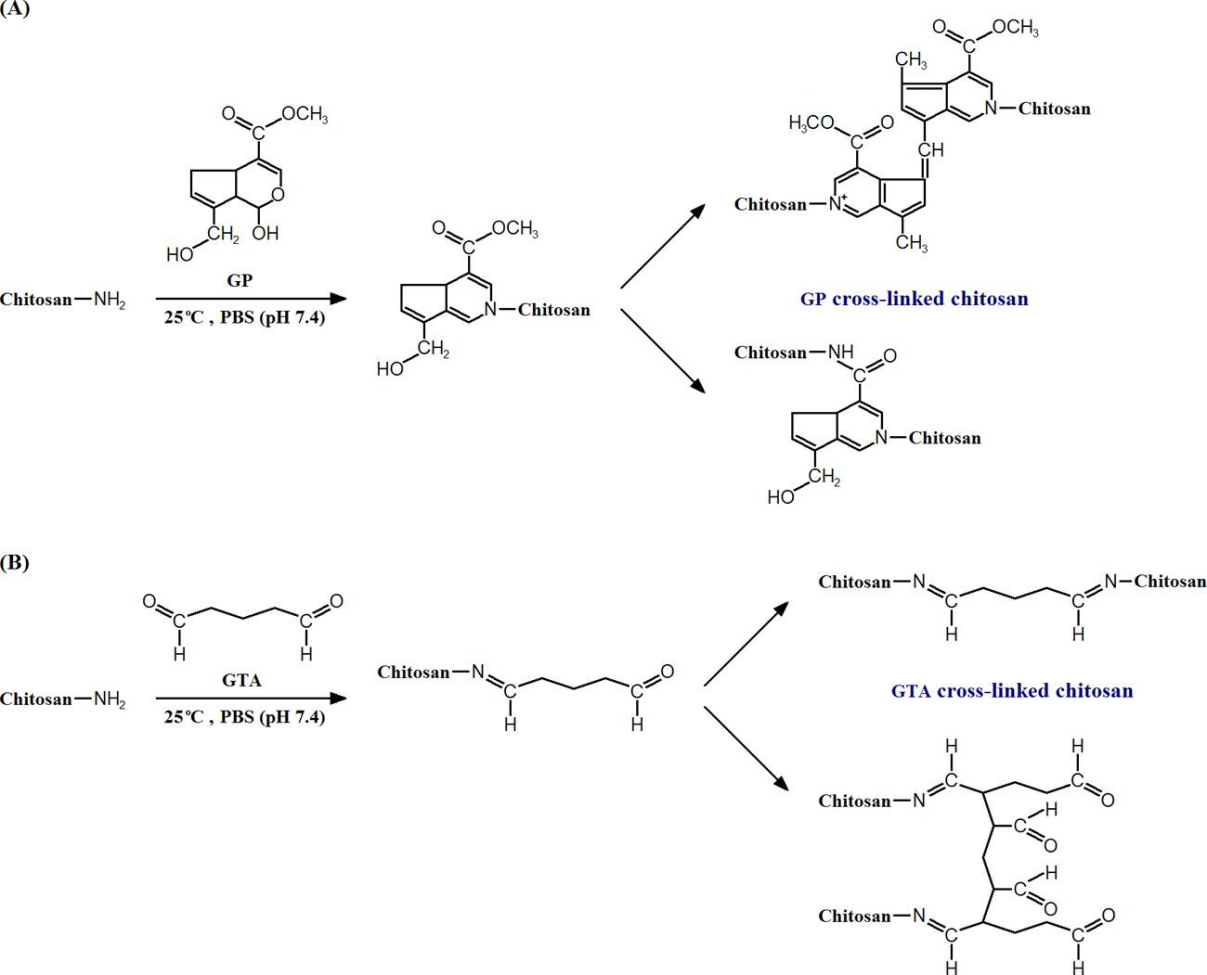
Cross-linking reaction scheme of chitosan with (**A**) genipin (GP), and (**B**) glutaraldehyde (GTA).

In this study, to cross-link the chitosan materials, we adopted a film immersion method as previously described for the chemical modification of gelatin sheets [23] and human amniotic membranes [13]. The general reaction mechanisms for the GP and GTA cross-linking of chitosan are presented in Figure 1A and B, respectively. The formation of GP-amino group monomer is achieved by nucleophilic attack of free amino groups of chitosan on the third carbon of GP. Then, cross-linking is carried out by either radical polymerization of the monomers or S_N_2 nucleophilic substitution reaction involving the replacement of the ester group on the monomer with a secondary amide linkage. The detailed reaction pathways for GP cross-linking of amino group containing biopolymers has been discussed by Butler and colleagues [24]. On the other hand, GTA can react with free amino groups of chitosan, leading to the formation of either the Schiff bases or the Michael-type adducts with terminal aldehydes.

**Figure 2.**
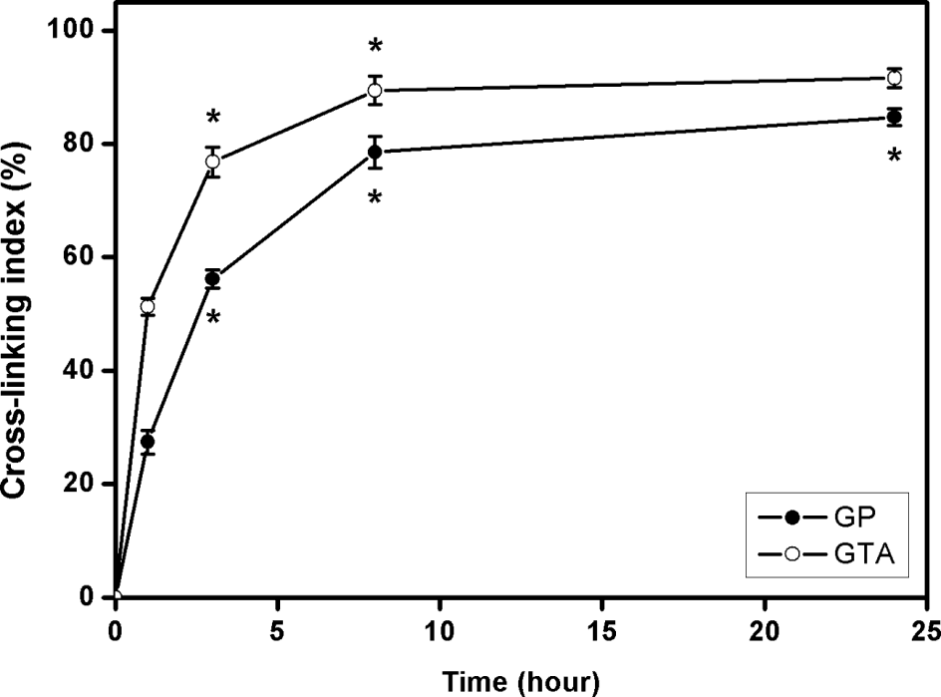
Cross-linking index of GP and GTA treated chitosan membranes as a function of cross-linking time. An asterisk indicates statistically significant differences (*p < 0.05; *n* = 5) for the mean value of cross-linking index compared with the value at the previous time point.

The ninhydrin assay has been shown to be applicable for determining the extent of cross-linking in amino group containing biomaterials [4,13]. In this study, the cross-linking index of GP and GTA treated chitosan membranes was investigated as a function of cross-linking time (Figure 2). For GP-chi groups, the cross-linking degree significantly increased with increasing reaction time from 1 to 24 h (*p* < 0.05). A similar trend was found for the GTA-chi groups. At each time point, the cross-linking index of chitosan samples treated with GTA was significantly higher than their counterparts with GP (*p* < 0.05), which indicated that the GTA demonstrates a higher reactivity during chemical cross-linking. These findings are in accordance with previous results showing that the GTA produces stronger cross-linking to gelatin molecules than using GP [25]. It is known that the cross-linker concentration, pH, temperature, and time greatly affect the cross-linking degree of biomaterials [26], which may further determine their compatibility with cells and tissues. Here, the chitosan membranes were modified with 10 mM of either GP or GTA at 25 °C, pH 7.4 for different time periods (0–24 h). To examine the influence of chemical cross-linkers on the biocompatibility of chitosan materials, the GP and GTA treated samples with the same extent of cross-linking (*i.e.*, 78.5 ± 2.8% for GP-chi at 8 h and 76.8 ± 2.6% for GTA-chi at 3 h) were characterized by various *in vitro* assays.

**Figure 3.**
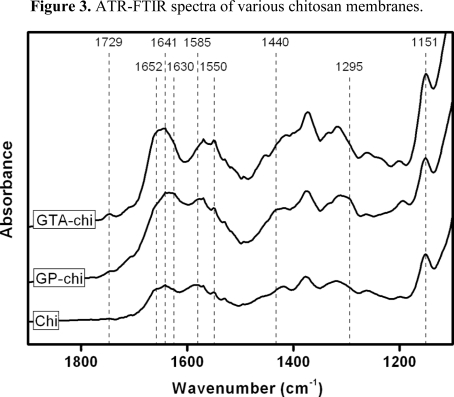
ATR-FTIR spectra of various chitosan membranes.

Before cytocompatibility tests, both above-mentioned samples with the same cross-linking degree were studied by Attenuated total reflection-Fourier transform infrared (ATR-FTIR) spectroscopy, which is a useful tool for the identification of molecular interactions in the chemically modified biomaterials [13]. In this paper, it is difficult to interpret the spectra, primarily due to the large extent of hydrogen bonding in chitosan. Therefore, representative spectra for various membranes in the wavenumber range of 1900–1100 cm^−1^ are shown in Figure 3. For Chi groups, the samples had several absorption bands at 1641 cm^−1^ (C=O stretching in amide I), 1550 cm^−1^ (N-H bending in amide II), 1585 cm^−1^ (N-H bending in non-acetylated 2-aminoglucose primary amine), and 1151 cm^−1^ (C-O-C asymmetric stretching), which are characteristic of chitosan polysaccharide structure [27]. After GP cross-linking, the membranes revealed additional peaks at 1295, 1440, and 1630 cm^−1^, which were assigned to the C-O-C asymmetric stretching and the CH_3_ bending of the methyl ester, and C=C ring stretching, respectively. These data are in agreement with previous FTIR study on GP treated biopolymers [28]. The spectra of the GTA-chi groups exhibited absorption at 1652 cm^−1^, indicating the presence of C=N bonds from the Schiff bases. Furthermore, a new peak appeared at 1729 cm^−1^ which, according to the literature [29], corresponded to the carbonyl groups in the Michael-type adducts.

### 2.2. Cell Viability Assays

**Figure 4.**
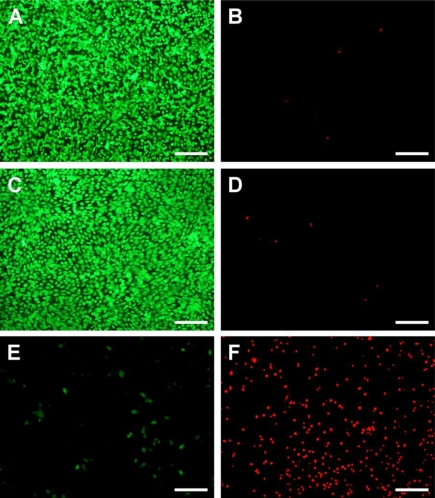
Cell viability of ARPE-19 cultures was determined by staining with Live/Dead Viability/Cytotoxicity Kit in which the live cells fluoresce green and the dead cells fluoresce red. Green (**A**, **C**, **E**) and red (**B**, **D**, **F**) fluorescence images of cells after exposure to 5 mg of different types of chitosan membranes (A, B) Chi, (C, D) GP-chi, and (E, F) GTA-chi for 3 days at 37 °C. Scale bars indicate 100 μm.

**Figure 5.**
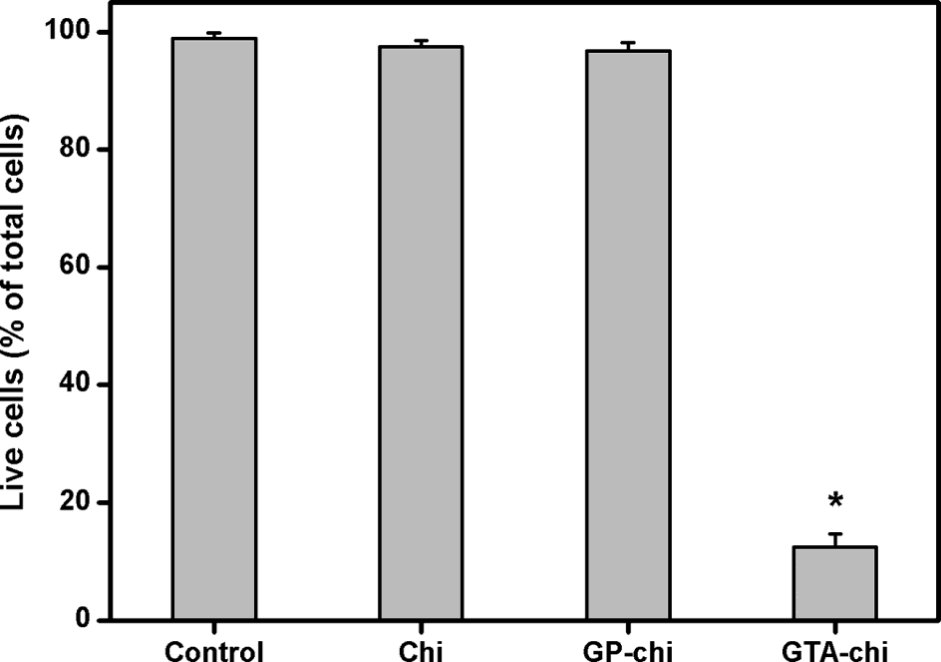
Mean percentage of live cells in the ARPE-19 cultures exposed to various chitosan membranes (5 mg) as measured by the Live/Dead assay. An asterisk indicates statistically significant differences (*p < 0.05; *n* = 3) as compared to controls (without materials).

Figure 4 shows representative photographs of ARPE-19 cells labeled with Live/Dead stain, where the live cells fluoresce green and the dead cells fluoresce red. Similar to our previous study [30], the cultures from the control groups maintain good viability (data not shown). A large percentage of live cells was also observed in the Chi and GP-chi groups (Figure 4A–D), which indicates that these chitosan samples had good cytocompatibility. However, in the GTA-chi groups, the cultures contained a large number of dead cells (Figure 4E and F). Quantitative analysis of cell viability was performed following the Live/Dead assay, and the results are shown in Figure 5. The mean percentage of live cells did not show a significant difference between the control, Chi, and GP-chi groups (*p* > 0.05) after three days in culture. In addition, the viability of these RPE cell lines was greater than 96%. In contrast, the mean percentage of live cells was significantly reduced by about 86% (*p* < 0.05) for GTA-chi as compared to those of the control groups. The ARPE-19 cells exposed to GTA cross-linked chitosan membranes had relatively higher cytotoxicity than did those to GP treated samples.

Chitosan is a well-known biocompatible and bioactive material and has been under investigation for a vast array of biomedical applications including sutures, wound dressings, tissue engineering scaffolds, and drug/gene delivery vehicles [31]. Several reports have demonstrated the biocompatibility of chitosan by using various *in vitro* and *in vivo* assays [32,33]. The present study, based on cell viability data, provides evidence to support these earlier findings. Although chitosan membrane has no toxic effects on RPE cells *in vitro*, chemical cross-linking may induce cell/tissue responses to biomaterials. As shown in the literature, GTA has been used to modify different biopolymers such as gelatin [4], hyaluronic acid [16], and chitosan [19] for creating intermolecular covalent bonds. However, GTA cross-linking is associated with cytotoxicity, which may be due to the specific linkages present in the cross-linking structure [4,16]. In this paper, GTA may react with free amino groups of biomaterials to form Michael-type adducts with terminal aldehydes, thereby causing harmful irritation to cultured cells. On the contrary, the chitosan molecules bridged by GP are well tolerated with negligible toxicity. Mi *et al.* [19] have reported that the human foreskin fibroblasts grow with a significantly higher viability on GP cross-linked chitosan films *versus* GTA treated counterparts. Consistent with these data, we showed that the chitosan materials cross-linked with GP, in contrast to those with GTA, are more compatible toward human RPE cells and have potential suitability for application as drug carriers in the treatment of posterior segment diseases.

### 2.3. Pro-Inflammatory Gene and Cytokine Expression Analyses

**Figure 6.**
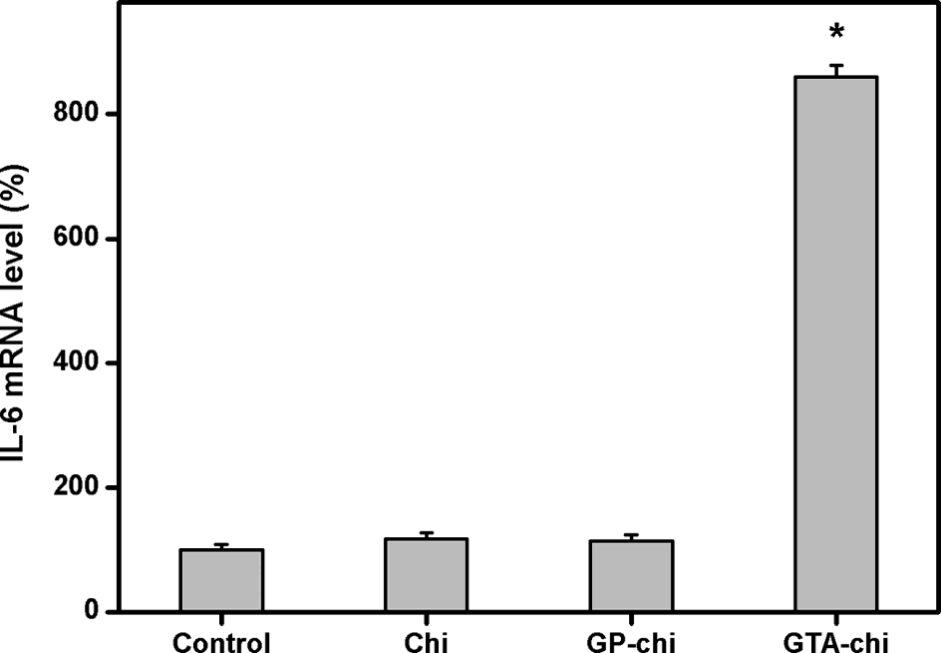
Gene expression of IL-6 in ARPE-19 cells incubated with various chitosan membranes (5 mg) for 3 days, measured by real-time RT-PCR. Normalization was done using GAPDH. Data in the experimental groups are percentages relative to that of control groups (without materials). An asterisk indicates statistically significant differences (*p < 0.05; *n* = 3) as compared to controls.

**Figure 7.**
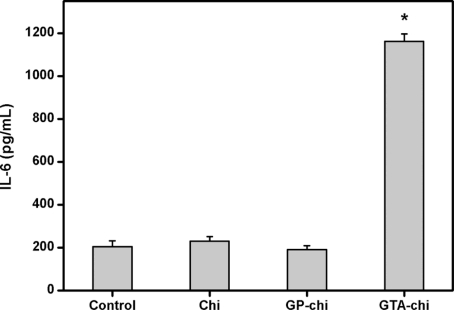
Level of IL-6 released from ARPE-19 cell cultures after incubation with various chitosan membranes (5 mg) for 3 days. An asterisk indicates statistically significant differences (*p < 0.05; *n* = 4) as compared to controls (without materials).

IL-6, which appears to be a major cytokine-triggering mediator in the later part of the acute phase, can be used as an indicator of biocompatibility of the materials tested [34]. For evaluation of inflammatory responses to cross-linked chitosan membranes, we utilized real-time RT-PCR and ELISA to quantify the IL-6 expression at both the mRNA and protein levels. As shown in Figure 6, the pro-inflammatory gene expression of ARPE-19 cells after three days of exposure to biomaterials from the Chi, GP-chi, and GTA-chi groups was 117.4 ± 9.5, 113.8 ± 10.2, 860.3 ± 17.9%, respectively. A significant up-regulation of IL-6 mRNA expression was observed in the GTA-chi groups compared with the other groups (*p* < 0.05). On the other hand, Figure 7 shows the ARPE-19 cell secretion of IL-6 in response to various chitosan samples. The measured concentration of IL-6 in the GTA-chi groups was 1161.7 ± 34.4 pg/mL, which was significantly higher than those in the control (204.8 ± 27.2 pg/mL), Chi (229.3 ± 21.6 pg/mL), and GP-chi (190.5 ± 18.1 pg/mL) groups (*p* < 0.05).

Chatelet *et al.* [35] have reported that the chitosan-based biomaterials do not show any inflammatory or allergic reaction following implantation, injection, topical application or ingestion in the human body. Later studies from Mi *et al.* [9] and VandeVord *et al.* [32] also demonstrated that the tissue near the implantation site revealed little pathological inflammatory responses to intramuscular chitosan microspheres and intraperitoneal/subcutaneous chitosan scaffolds. The results of the present study on the degrees in inflammatory reaction confirm the *in vitro* ocular biocompatibility of chitosan materials. However, our data also show that the GTA cross-linked chitosan membranes have a marked stimulatory influence on IL-6 production. More recently, we investigated the pro-inflammatory cytokine expression elicited in ARPE-19 cells in response to GTA cross-linked gelatin hydrogels and found similar effects [4]. The treatment of these samples with glycine following chemical modification may partially decrease cytotoxicity and inhibit inflammation, implying the role of aldehyde groups of the cross-linker on the interaction with RPE cells. In contrast, the ARPE-19 cells exposed to GP cross-linked chitosan materials demonstrated slightly lower levels of IL-6 than did those to non-cross-linked counterparts, although these differences were not significant (*p* > 0.05). It is known that GP is a naturally occurring cross-linker and has been widely applied in herbal medicine and food industry [26]. Koo *et al.* [36] have reported that the GP possesses anti-inflammatory activities and is a specific hydroxyl radical scavenger. This may explain our results showing that GP cross-linked chitosan materials caused minimal cellular inflammatory reaction.

### 2.4. Apoptosis Assays

**Figure 8.**
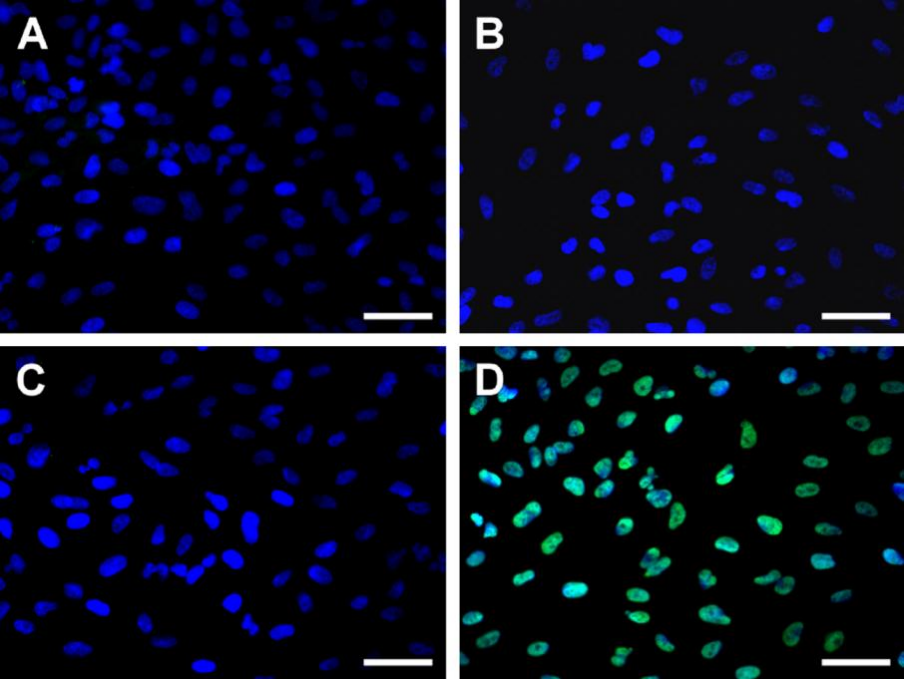
Apoptotic cells labeled with TUNEL assay in the ARPE-19 cultures. Fluorescence micrographs of control cells (without materials) (**A**), and cells after exposure to 5 mg of different types of chitosan membranes (**B**) Chi, (**C**) GP-chi, and (**D**) GTA-chi for 24 h at 37 °C. Blue fluorescence is DAPI nuclei staining. Green fluorescence is TUNEL-positive nuclei staining. Scale bars indicate 30 μm.

**Figure 9.**
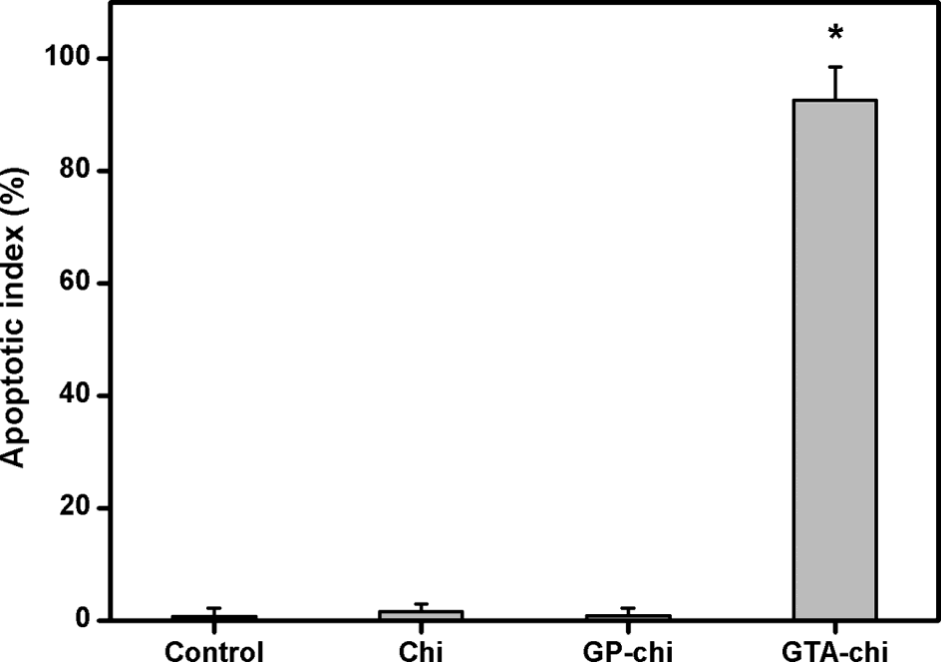
Apoptotic index of ARPE-19 cells exposed to various indicated chitosan membranes (5 mg) as determined by the TUNEL assay. An asterisk indicates statistically significant differences (*p < 0.05; *n* = 3) as compared to controls (without materials).

Figure 8 shows TUNEL analysis of the ARPE-19 cells after exposure to various chitosan membranes for 24 h. While TUNEL-positive cells were observed in the DNase-treated cultures (positive controls), no apoptotic activity was detectable in the negative controls (data not shown). Representative fluorescence microscopic images of the control, Chi and GP-chi groups demonstrated total cell nuclei stained with DAPI in blue (Figure 8A–C). In contrast, numerous TUNEL-labeled cells with fragmented DNA (green) could be visualized within the cultures from the GTA-chi groups (Figure 8D). The apoptotic index determined by TUNEL assay is shown in Figure 9. There was no statistically significant difference in the apoptotic index between the control, Chi, and GP-chi groups (*p* > 0.05). In the case of Chi and GP-chi groups, only approximately 1% of the cells were positively labeled with TUNEL. These results indicate that the non-cross-linked and GP cross-linked chitosan materials do not cause apoptosis in RPE cells. The proportion of apoptotic cells was significantly higher in cultures treated with GTA cross-linked chitosan membranes compared with those exposed to GP cross-linked samples (*p* < 0.05), suggesting that cross-linking of biomaterials with GTA may be relevant for apoptosis induction.

Our group recently developed an intraocular cell delivery system using biopolymer-based carriers [15,16,37–39]. Since biocompatibility is a prerequisite for this carrier implant, an *in vivo* animal model was previously established to assess the inflammatory response of the eye to biomaterials [40]. Here, we investigated the *in vitro* cellular responses to various chitosan materials using ARPE-19 cultures before further *in vivo* animal experiments. The results of both cell viability assays and pro-inflammatory gene and cytokine expression analyses indicate that the GP cross-linked chitosan membranes have good compatibility towards human RPE cells. On the contrary, the samples cross-linked with GTA show cytotoxicity and are not suitable for intraocular application. It has been reported that cross-linker toxicity to osteoblasts cultured on GTA treated collagen/poly(vinyl alcohol) films is by the mechanism of apoptosis [41]. In accordance with these earlier observations, our data imply that the programmed cell death may be caused by the interaction of the exposed aldehyde groups with the surface of ARPE-19 cells. A recent study from Marczak *et al.* [42] also demonstrated that GTA would produce significant perturbations in the organization of plasma membrane lipids in human erythrocytes, thereby leading to conformational alterations in membrane cytoskeletal proteins and apoptosis (self destruction) of the cells. In this work, the GTA cross-linked chitosan materials triggered-apoptosis at an early stage may induce toxicity in the RPE cells later.

## 3. Experimental Section

### 3.1. Materials

Chitosan (Cat. No. 50494), derived from crab shell, was a commercial powder supplied by Fluka (Milwaukee, WI, U.S.). According to information from the manufacturer, the chitosan samples used as raw materials had a degree of deacetylation of 95–98% and a molecular weight of approximately 150 kDa. Glutaraldehyde and ninhydrin reagent were purchased from Sigma-Aldrich (St. Louis, MO, U.S.). Genipin was purchased from Wako Pure Chemical Industries (Osaka, Japan). Phosphate-buffered saline (PBS, pH 7.4) was obtained from Biochrom AG (Berlin, Germany). Dulbecco’s modified Eagle’s medium/Ham’s F12 nutrient mixture (DMEM/F12) and TRIzol reagent were purchased from Gibco-BRL (Grand Island, NY, U.S.). Fetal bovine serum (FBS) and the antibiotic/antimycotic (A/A) solution (10,000 U/mL penicillin, 10 mg/mL streptomycin and 25 μg/mL amphotericin B) were obtained from Biological Industries (Kibbutz Beit Haemek, Israel). All the other chemicals were of reagent grade and used as received without further purification.

### 3.2. Preparation of GP or GTA Cross-Linked Chitosan Membranes

The chitosan membranes (Chi group) were prepared by solution casting method. In brief, chitosan (1 g) was added to 1% v/v aqueous acetic acid (50 mL) with stirring until complete dissolution. To remove insoluble substance, the solution was passed through a filter paper (Tokyo Roshi Kaisha, Tokyo, Japan). Then, 0.5 mL of chitosan solution was poured in a well of a 24-well plate (Falcon, Becton Dickinson Labware, Franklin Lakes, NJ, U.S.) and air-dried for 2 days at 25 °C to obtain membranes (about 10 μm thick). The membrane samples were incubated in a 0.5 N NaOH solution for 1 h and washed extensively with deionized water until neutrality.

The chitosan materials were treated with GP (GP-chi group) or GTA (GTA-chi group) by respectively immersing the membrane samples in 5 mL of PBS containing 10 mM cross-linker. The cross-linking reaction was allowed to proceed at 25 °C for different time periods (0–24 h). To eliminate the residual GP or GTA, the cross-linked membranes were washed extensively in deionized water. Then, the samples were dried *in vacuo* for 24 h and sterilized in a graded series of ethanol solutions and thoroughly rinsed in sterilized PBS for use in the *in vitro* experiments.

The amount of free amino groups of chitosan membranes was determined to evaluate their extent of cross-linking [4]. The test sample was weighed and heated with a ninhydrin solution for 20 min. After the test solution was cooled to room temperature and diluted in 95% ethanol, the optical absorbance of the solution was recorded with a UV-visible spectrophotometer (Thermo Scientific, Waltham, MA, U.S.) at 570 nm using glycine at various known concentrations as standard. The amount of free amino groups in the chitosan materials before (*C*_b_) and after (*C*_a_) cross-linking is proportional to the optical absorbance of the solution. The extent of cross-linking of the chitosan membranes was calculated as cross-linking index (%) = ((*C*_b_ − *C*_a_)/*C*_b_) × 100. Results were averaged on five independent runs.

The FTIR spectroscopy of various chitosan membranes was performed using a FT-730 ATR-FTIR Spectrophotometer (Horiba, Japan) according to the previously published method [13]. The spectra were recorded between 1900 and 1100 cm^−1^ with a resolution of 4 cm^−1^.

### 3.3. Human RPE Cell Line Cultures

ARPE-19 cells, a spontaneously immortalized human cell line (BCRC No. 60383) with morphological and functional characteristics similar to adult human RPE [30], were purchased from the Bioresource Collection and Research Center (Hsinchu, Taiwan, ROC). The cells were maintained in regular growth medium containing DMEM/F12, 10% FBS, and 1% A/A solution. Cultures were incubated in a humidified atmosphere of 5% CO_2_ at 37 °C. The cells from passage 38 were used for experiments.

For evaluation of cellular responses to cross-linked chitosan materials, an indirect contact methodology was used as described elsewhere [34]. In brief, ARPE-19 cells (7 × 10^4^ cells/well) were seeded in 24-well plates and maintained at 37 °C with 5% CO_2_. Using cell culture inserts (Falcon 3095, Becton Dickinson Labware, Franklin Lakes, NJ, U.S.), each well of a 24-well plate was divided into two compartments. A 1-cm^2^ membrane sample (5 mg) was then placed into the inner well of the double-chamber system to examine the cells cultured on the plastic plate. At specific time intervals, the qualitative and quantitative assays were performed following removal of the inserts and chitosan samples. ARPE-19 cells in regular growth medium without test materials served as control groups.

### 3.4. Cell Viability Assays

ARPE-19 cells were grown to confluence and then were exposed to various chitosan membranes for 3 days. Cell viability was determined using the Live/Dead Viability/Cytotoxicity Kit from Molecular Probes (Eugene, OR, U.S.) [43]. This assay uses intracellular esterase activity to identify the living cells; the process cleaves the calcein acetoxymethyl to produce a green fluorescence. Ethidium homodimer-1 can easily pass through the damaged cell membranes of dead cells to bind to the nucleic acids, yielding a red fluorescence. After washing three times with PBS, the cultures were stained with a working solution consisting of 2 μL of ethidium homodimer-1, 1 mL of PBS, and 0.5 μL of calcein acetoxymethyl. By fluorescence microscopy (Axiovert 200M; Carl Zeiss, Oberkochen, Germany), three different areas each containing approximately 500 cells were counted at 100× magnification. All experiments were performed in triplicate, and the viability of the ARPE-19 cell cultures was expressed as the average ratio of live cells to the total number of cells in these nine different areas.

### 3.5. Pro-Inflammatory Gene and Cytokine Expression Analyses

ARPE-19 cells were grown to confluence and then were exposed to various chitosan membranes for 3 days. Interleukin-6 (IL-6) expression was detected at both messenger RNA (mRNA) and protein levels [30]. Total RNA was isolated from ARPE-19 cells with TRIzol reagent according to the manufacturer’s procedure. Reverse transcription of the extracted RNA (1 μg) was performed using ImProm-II (Promega, Madison, WI, U.S.) and Oligo(dT)_15_ primers (Promega). The primers used to amplify the human IL-6 complementary DNA (cDNA) were 5′-CCACTCACCTCTTCAGAACGAA-3′ (sense) and 5′-GGCAAGTCTCCTCATTGAATCC-3′ (antisense), and those used to amplify the internal control cDNA, glyceraldehyde-3-phosphate dehydrogenase (GAPDH), were 5′-TGGTATCGTGGAAGGACTCATGAC-3′ (sense) and 5′-ATGCCAGTGAGCTTCCCGTTCAGC-3′ (antisense). Quantitative real-time reverse transcription polymerase chain reaction (RT-PCR) was performed on a Light-Cycler instrument (Roche Diagnostics, Indianapolis, IN, U.S.) according to the manufacturer’s instructions with FastStart DNA Master SYBR Green I reagent (Roche Diagnostics). Each sample was determined in triplicate and the results for IL-6 were normalized to the level of GAPDH mRNA. On the other hand, aliquots of the supernatant were collected to measure the IL-6 levels. The release of IL-6 from cultivated cells into the conditioned medium was detected by the Quantikine enzyme-linked immunosorbent assay (ELISA) kit (R&D Systems, Minneapolis, MN, U.S.) specific for human IL-6. Cytokine bioassays were performed according to the manufacturer’s instructions. Photometric readings at 450 nm were measured using the Spectrophotometer (ThermoLabsystems, Vantaa, Finland). Results were expressed as pg/mL. All experiments were conducted in quadruplicate.

### 3.6. Apoptosis Assays

After seeding overnight, the attached RPE cells were exposed to various chitosan membranes for 24 h. Apoptotic cells in ARPE-19 cultures were determined using a terminal deoxynucleotidyl transferase (TdT)-mediated dUTP nick end labeling (TUNEL) assay (Roche Diagnostics). Samples were fixed with 4% paraformaldehyde for 1 h at room temperature. After washing with PBS, the fixed specimens were permeabilized in 0.1% Triton X-100 in 0.1% sodium citrate for 2 min on ice and incubated with a mixture of TdT solution and fluorescein isothiocyanate dUTP solution in a humidified chamber for 1 h at 37 °C. The negative controls were incubated with distilled water in place of TdT enzyme. Cells treated with DNase served as positive controls. To visualize cell nuclei, the samples were stained with 4′,6-diamidino-2-phenylindole (DAPI; Vector, Peterborough, England) and examined microscopically at 400× magnification. Ten different areas were randomly selected, and the number of TUNEL-positive cell nuclei was quantified. All experiments were conducted in triplicate, and the apoptotic index was calculated as the average ratio of apoptotic cells to the total number of cells in these 30 different areas.

### 3.7. Statistics

Results were expressed as mean ± standard deviation. Comparative studies of means were performed using one-way analysis of variance (ANOVA). Significance was accepted with *p* < 0.05.

## 4. Conclusions

In this work, cell viability assays, cytokine expression analyses, and apoptosis assays were performed to examine the interaction between human RPE cells and various chitosan materials. The biocompatibility of non-cross-linked chitosan membranes is demonstrated by the absence of any signs of toxicity or inflammation reaction. We have also investigated the effect of cross-linker functionality on the *in vitro* cellular responses to chemically modified chitosan samples. The results suggest that while the chitosan molecules bridged by GP are satisfactorily cytocompatible, the counterparts treated by GTA do not seem to be tolerated. In terms of material safety, the GP cross-linked chitosan may have biocompatibility for potential application as delivery carrier in the treatment of posterior segment diseases.
